# Prevalence of *pfhrp2* and *pfhrp3* gene deletions in Puerto Lempira, Honduras

**DOI:** 10.1186/s12936-014-0537-7

**Published:** 2015-01-21

**Authors:** Joseph F Abdallah, Sheila Akinyi Okoth, Gustavo A Fontecha, Rosa Elena Mejia Torres, Engels I Banegas, María Luisa Matute, Sandra Tamara Mancero Bucheli, Ira F Goldman, Alexandre Macedo de Oliveira, John W Barnwell, Venkatachalam Udhayakumar

**Affiliations:** Malaria Branch, Division of Parasitic Diseases and Malaria, Center for Global Health, Centers for Disease Control and Prevention, 1600 Clifton Road, MS D-67, Atlanta, GA 30333 USA; IHRC, Inc., Atlanta, GA USA; Atlanta Research and Education Foundation, Decatur, GA USA; Microbiology Research Institute, National Autonomous University of Honduras, Tegucigalpa, Honduras; Pan American Health Organization, Tegucigalpa, Honduras; National Malaria Laboratory, National Department of Surveillance, Ministry of Health, Tegucigalpa, Honduras; National Malaria Program, Department of Health Promotion, Ministry of Health, Tegucigalpa, Honduras

**Keywords:** *Plasmodium falciparum*, Histidine-rich protein, Rapid diagnostic tests, Microsatellites, Honduras

## Abstract

**Background:**

Recent studies have demonstrated the deletion of the histidine-rich protein 2 (PfHRP2) gene (*pfhrp2*) in field isolates of *Plasmodium falciparum*, which could result in false negative test results when PfHRP2-based rapid diagnostic tests (RDTs) are used for malaria diagnosis. Although primary diagnosis of malaria in Honduras is determined based on microscopy, RDTs may be useful in remote areas. In this study, it was investigated whether there are deletions of the *pfhrp2*, *pfhrp3* and their respective flanking genes in 68 *P. falciparum* parasite isolates collected from the city of Puerto Lempira, Honduras. In addition, further investigation considered the possible correlation between parasite population structure and the distribution of these gene deletions by genotyping seven neutral microsatellites.

**Methods:**

Sixty-eight samples used in this study, which were obtained from a previous chloroquine efficacy study, were utilized in the analysis. All samples were genotyped for *pfhrp2*, *pfhrp3* and flanking genes by PCR. The samples were then genotyped for seven neutral microsatellites in order to determine the parasite population structure in Puerto Lempira at the time of sample collection.

**Results:**

It was found that all samples were positive for *pfhrp2* and its flanking genes on chromosome 8. However, only 50% of the samples were positive for *pfhrp3* and its neighboring genes while the rest were either *pfhrp3*-negative only or had deleted a combination of *pfhrp3* and its neighbouring genes on chromosome 13. Population structure analysis predicted that there are at least two distinct parasite population clusters in this sample population. It was also determined that a greater proportion of parasites with *pfhrp3*-(and flanking gene) deletions belonged to one cluster compared to the other.

**Conclusion:**

The findings indicate that the *P. falciparum* parasite population in the municipality of Puerto Lempira maintains the *pfhrp2* gene and that PfHRP2-based RDTs could be considered for use in this region; however continued monitoring of parasite population will be useful to detect any parasites with deletions of *pfhrp2*.

## Background

Honduras has the highest burden of malaria and the highest proportion of *Plasmodium falciparum* cases in Central America [1,2], but is steadily progressing toward malaria elimination. The number of malaria cases per year in the country has dropped from approximately 35,000 in 2000 to approximately 6,400 cases in 2012 [3]. Less than 10% of malaria cases in Honduras are caused by *P. falciparum*. Malaria diagnosis in the country is primarily conducted by microscopic examination of Giemsa-stained thick and thin blood smears. In recent years, however, malaria rapid diagnostic tests (RDTs) have become valuable tools for use in remote areas where microscopy use may not be feasible or where microscopy results would not be immediately available.

Malaria RDTs are immunochromatographic tests that detect malaria parasite antigens. The majority of malaria RDTs detect histidine-rich protein 2 (PfHRP2), which is produced during the asexual blood stage of the *P. falciparum* life cycle but not by other human malaria parasite species, making PfHRP2-based RDTs species-specific [4]. Malaria parasite lactate dehydrogenase (pLDH) and aldolase are two other targets of some commercial malaria RDTs; although these tests are mostly pan-specific, recent modifications have improved their ability to differentiate species with pLDH based RDTs. Some PfHRP2-detecting RDTs are known to cross-react with PfHRP3 [5]. PfHRP2 and PfHRP3 are structural homologs [6], but their respective genes are located on different chromosomes, with *pfhrp2* found on chromosome 8 and *pfhrp3* on chromosome 13.

Recently, it was reported that approximately 40% of *P. falciparum* field isolates in the Peruvian Amazon lacked *pfhrp2*. Furthermore, 70% of these parasites had deleted *pfhrp3* and 21.6% of them were negative for both *pfhrp2* and *pfhrp3* [7]. Although such large scale deletion of *pfhrp2* has not been reported in other parts of the world, occurrence of a few *pfhrp2*-negative parasites has been reported in Mali [8], Senegal [9] and India [10].

Little is known about the extent of *pfhrp2* and *pfhrp3* deletions in *P. falciparum* parasites from Honduras, which has the highest burden of malaria in Central America [3]. It was important to monitor if *pfhrp2*-negative parasites were present in Honduran *P. falciparum* populations because of the implications for the use of malaria RDTs in this country for case management and malaria elimination programs. The aim of this study was to determine whether there was deletion of *pfhrp2*, *pfhrp3*, and their respective flanking genes in natural *P. falciparum* parasites collected in Honduras and to investigate the possible effect of parasite population structure on the distribution of these gene deletions.

## Methods

### Study site and sample collection

Sixty-eight *P. falciparum* samples that were collected during a previous study conducted between September 2008 and September 2009 in the city of Puerto Lempira, which is located in the province of Gracias a Dios, Honduras, were available for this retrospective investigation [11]. In the original study, filter paper blood spots were collected from febrile patients between the ages of six months and 60 years who had uncomplicated *P. falciparum* mono-infection and had provided written informed consent at the time of enrollment. These patients participated in a clinical trial to assess the efficacy of chloroquine. Further information on the patients and study has been published previously [11]. The study was approved by the institutional ethical review committee of the Ethics Committee of the Medical Sciences Faculty of the National Autonomous University of Honduras (UNAH-IRB 00003070). Investigators from the Centers for Disease Control and Prevention obtained institutional permission to use these samples for the current study under a non-research determination.

### Extraction of parasite DNA

Genomic DNA was extracted from dried blood spots on filter paper using the Qiagen™ kit (QIAGEN, Valencia CA) according to the manufacturer’s instructions and resuspended in 150 μl elution buffer.

### Confirmation of *P. falciparum* infection by PCR

In order to determine if there were sufficient quantities and quality of DNA present in the samples collected, two genes were amplified, 18s ribosomal RNA (*18S rRNA*) and merozoite surface protein 2 (*msp2*), using established PCR protocols [12]. An assumption was made that the successful amplification of both genes was indicative of a reasonable quantity and quality of DNA that would allow for the amplification of *pfhrp2*, *pfhrp3* and their respective neighbouring genes. Therefore, only those samples that successfully amplified for these two genes were included for final analysis of *pfhrp2* and *pfhrp3* gene deletion. The PCR amplification of the 18S ribosomal RNA gene was performed using methods previously described [12]. Primer sequences for this nested reaction were as follows: 5′CCT GTT GTT GCC TTA AAC TTC3′ and 5′TTA AAA TTG TTG CAG TTA AAA CG3′ for the primary reaction and 5′ TTA AAC TGG TTT GGG AAA ACC AA ATA TAT T 3′ and 5′ ACA CAA TGA ACT CAA TCA TGA CTA CCC GTC 3′ for the secondary reaction. Briefly, PCR reactions were performed in 25 μl total volume containing 10× buffer, 4 mM MgCl2, 200 μM dNTPs, 250 nM primers, 1.25 units of Taq Polymerase (New England Biolabs, Ipswich, MA, USA), and 1–3 μl of DNA template. After confirmation of *Plasmodium* genus, species-specific primers were used to confirm *P. falciparum* infection.

The *msp2* gene was amplified using a previously described method [13]. Briefly, a nested PCR reaction was used in which primary amplification targeted a conserved region of this gene and a secondary PCR step that amplified a polymorphic region of *msp2* (3D7 and FC27 families). The primer sequences for the primary reaction were 5′GAA GGT AAT TAA AAC ATT GTC 3′ and 5′GAT GTT GCT GCT CCA CAG3′ and 5′GAG TAT AAG GAG AAG TAT G3′ and 5′CTA GAA CCA TGA ATA TGT CC3′ for the secondary reaction. Confirmatory PCR reactions for *msp2* were performed in 20 μl total volume containing 10× buffer with MgCl2, 200 μM dNTPs, 250 nM primers, 1.25 units of Taq Polymerase (New England Biolabs, Ipswich, MA, USA), and 1–3 μl of DNA template. When PCR amplification was not successful for either gene, it was repeated to confirm the result. However, if the first two PCR amplification results were discordant, the amplification was performed a third time and the two concordant results were scored as the final result.

### Detection of *pfhrp2*, *pfhrp3* and flanking genes by PCR

**Figure 1:**
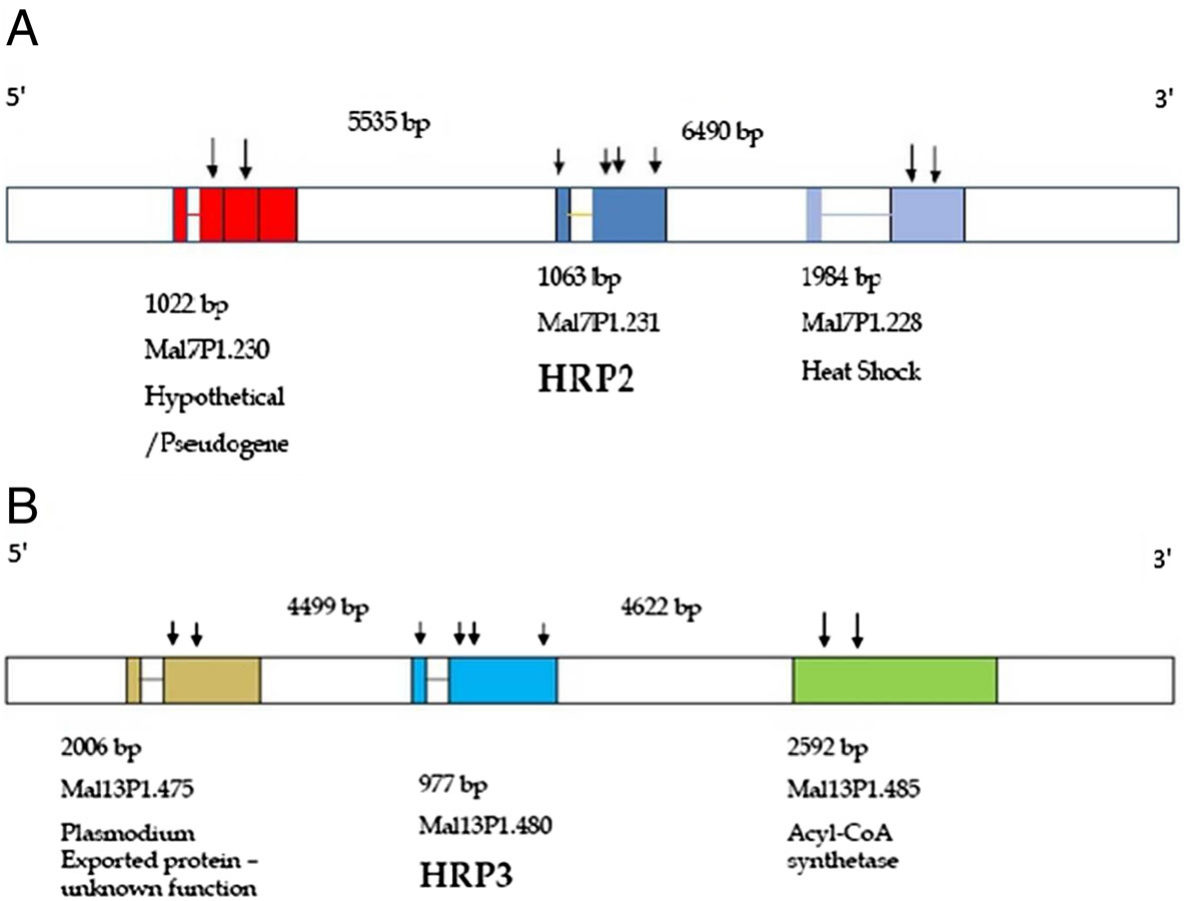
**Schematic of (A)** ***Pfhrp2*** **and (B)** ***Pfhrp3*** **and their respective flanking genes.** In PlasmoDB version 8.0, from which gene information was obtained, *pfhrp2* was located on chromosome 7 although in a recent update of PlasmoDB this gene and its flanking genes were reassigned to Chromosome 8. *Pfhrp3* was reported to be immediately flanked by the indicated genes, all of which are on Chromosome 13. Arrows indicate the gene fragments that were amplified.

Nested PCR amplification of each of the fragments spanning exon 1, the intron, and exon 2 of *pfhrp2* and *pfhrp3* (Figure 1) were performed using the primers and reaction conditions described in Table 1. The primers listed in Table 1 were designed around highly conserved regions flanking each of the genes of interest in order to minimize the possibility of variation in amplification due to gene polymorphisms. Genes immediately flanking *pfhrp2* upstream (MAL7P1.230) and downstream (MAL7P1.228), and those flanking *pfhrp3* upstream (MAL13P1.475) and downstream (MAL13P1.485), were also amplified using the primers and reaction conditions described in Table 1.

**Table 1:** **Primer sequences and PCR reaction conditions to amplify** ***pfhrp2*** **,** ***pfhrp3*** **and their respective flanking genes**

**Gene**	**Reaction**	**Primer name**	**Primer sequence**	**Annealing temperature, X (°C)**	**Expected amplicon size (bp)**
PF3D7_0831900 (MAL7P1.230)	Primary	230 F1	5′ GAT ATC ATT AGA AAA CAA GAG CTT AG 3′	63	301
		230R	5′ TAT CCA ATC CTT CCT TTG CAA CAC C 3′		
	Nested	230 F	5′ TAT GAA CGC AAT TTA AGT GAG GCA G 3′	65	
		230R	5′ TAT CCA ATC CTT CCT TTG CAA CAC C 3′		
PfHRP2 Exon 1–2, PF3D7_0831800	Primary	2E12F1	5′ GGT TTC CTT CTC AAA AAA TAA AG 3′	55	228
		2E12R1	5′ TCT ACA TGT GCT TGA GTT TCG 3′		
	Nested	2E12F	5′ GTA TTA TCC GCT GCC GTT TTT GCC 3′	62	
		2E12R	5′ CTA CAC AAG TTA TTA TTA AAT GCG GAA 3′		
PF3D7_0831700 (MAL7P1.228)	Primary	228 F	5′ AGA CAA GCT ACC AAA GAT GCA GGT G 3′	60	198
		228R	5′ TAA ATG TGT ATC TCC TGA GGT AGC 3′		
	Nested	228 F1	5′ CCA TTG CTG GTT TAA ATG TTT TAA G 3′	63	
		228R	5′ TAA ATG TGT ATC TCC TGA GGT AGC 3′		
PF3D7_1372100, (MAL13P1.485)	Primary	485 F	5′ TTG AGT GCA ATG ATG AGT GGA G 3′	60	241
		485R	5′ AAA TCA TTT CCT TTT ACA CTA GTG C 3′		
	Nested	485 F1	5′ GTT ACT ACA TTA GTG ATG CAT TC 3′	59	
		485R	5′ AAA TCA TTT CCT TTT ACA CTA GTG C 3′		
PfHRP3 Exon 1–2, PF3D7_1372200	Primary	3E12F1	5′ GGT TTC CTT CTC AAA AAA TAA AA 3′	53	225
		3E12R1	5′ CCT GCA TGT GCT TGA CTT TA 3′		
	Nested	3E12F	5′ ATA TTA TCG CTG CCG TTT TTG CT 3′	62	
		3E12R	5′ CTA AAC AAG TTA TTG TTA AAT TCG GAG 3′		
PF3D7_1372400 (MAL13P1.475)	Primary	475 F	5′ TTC ATG AGT AGA TGT CCT AGG AG 3′	55	212
		475R	5′ TCG TAC AAT TCA TCA TAC TCA CC 3′		
	Nested	475 F	5′ TTC ATG AGT AGA TGT CCT AGG AG 3′	61	
		475R1	5′ GGA TGT TTC GAC ATT TTC GTC G 3′		

All reactions were conducted using the following reaction conditions: 95°C/5 min – [95°C/30 sec; X/30 sec; 68°C/30 sec] × 30 cycles – 68°C/5 min – 4°C/∞. The old PlasmoDB gene IDs are included in parentheses below the current IDs.

*Pfhrp2, pfhrp3* and flanking gene amplifications were performed in 20 μl total volume consisting of 10× buffer with 15 mM MgCl2, 200 μM dNTPs, 15 μM forward and reverse primers, 0.69 units of Taq Polymerase (Roche, F. Hoffman-LaRoche Ltd, Basel, Switzerland), and 2 μl of DNA template. An *in vitro* cultured *P. falciparum* parasite isolate from the Amazon region of Peru was used as a positive control for all *pfhrp2*, *pfhrp3*, and flanking gene amplification experiments. Cultured parasite isolate Dd2 (5 ng/μL) was used as the negative control for all PCR experiments on *pfhrp2* and its flanking genes because this isolate lacks all three genes [14]. Dd2 was also used as a positive control for all the PCR experiments on *pfhrp3* and its flanking genes because it has retained these genes. Similarly, cultured parasite isolate HB3 (5 ng/μL) from Honduras was used as the negative control for all *pfhrp3* and flanking gene amplifications because all three genes are absent from this isolate [6]. Additionally, HB3 was used as a positive control for all PCR experiments on *pfhrp2* and its flanking genes because these genes were present in this isolate.

Expected PCR product sizes are indicated in Table 1. All PCR products were separated and visualized on a 2% agarose gel. When there was a positive reaction, this result was accepted without further repetition. When a negative test result was obtained, the amplification was repeated for confirmation. If the second result was concordant with the first, this was accepted as the final result. However, if the second result was discordant with the previous test result, the experiment was conducted a third time. The two matching results out of three were scored as the final result.

### Prevalence of *pfhrp2*, *pfhrp3* and flanking gene deletions

The prevalence of *pfhrp2*, *pfhrp3*, and flanking gene deletions was determined by dividing the number of isolates that had deleted the gene by the total number of isolates determined to be positive for both *18S rRNA* and *msp2*.

### Multilocus genotyping of parasite samples

To determine the population structure of *P. falciparum* parasite samples, we genotyped samples using seven neutral microsatellite markers used in previous studies [15-18]. Since only a limited amount of DNA was available from the samples, we performed whole-genome amplification of the samples using the Repli-G amplification kit (Qiagen, Valencia CA). The whole genome amplified samples were used for the amplification of the following neutral microsatellite loci: TA1 and TA109, both of which are located on chromosome 6; poly α (chromosome 4); PfPK2 (chromosome 12) and 2490 (chromosome 10); C2M34 (chromosome 2) and C3M69 (chromosome 3) loci. The amplification products were labeled with fluorescent dyes (FAM or HEX) and their sizes assayed on an Applied Biosystems 3130 xl sequencer. The fragments were then scored using GeneMapper software v.3.7 (Applied Biosystems, Foster City CA) with default microsatellite settings, where bands smaller than 500 relative fluorescence units (rfu) were defined as background. Samples for which we obtained no amplification in some loci were re-analysed to complete the microsatellite haplotype profiles. If a locus did not amplify after two rounds of PCR, the result was recorded as negative.

### Heterozygosity estimate

Overall parasite genetic diversity was examined by using neutral microsatellite data to calculate the microsatellite locus-specific heterozygosity and number of alleles per locus (*A*) in the Excel Microsatellite Toolkit. Any microsatellite locus that did not amplify was assigned a null value (350) for the purpose of these calculations. To determine locus-specific heterozygosity, we used the virtual heterozygosity estimate (*H*_E_), defined as *H*_E_ = [*n*/(*n* − 1)][1 − Σ*p*_*i*_^2^], where *n* is the number of isolates analysed and *p*_*i*_ is the frequency of the *i*-th allele in the population. *H*_E_ gives the average probability that a pair of alleles randomly obtained from the population is different and the values range between 0 and 1.

### Cluster analysis

To determine the population structure of *P. falciparum* isolates collected in Puerto Lempira, a Bayesian approach was used to infer the number of genetically related clusters (*K*) from the individual microsatellite profiles generated with the seven neutral microsatellites. Only samples that were determined to be singly infected (based on obtaining a single peak for each locus by neutral microsatellite analysis) were used for cluster analysis (N = 65). The calculation was performed using Structure v2.3.3 [19] in which the likelihood of finding between one and ten clusters in this population (*K* = 1 to *K* = 10) was tested. Twenty replicates of the clustering algorithm for each value of *K* were performed with a burn-in period of 10,000 iterations and 100,000 Markov Chain Monte Carlo replications, using the admixture model with correlated allele frequencies [20]. The most likely number of clusters was defined by calculating the Δ*K* value as described by Evanno *et al.* [21] and results from Structure were entered into the Structure Harvester program [22].

### Analysis of genetic distance

Population pairwise F_ST_ calculations were performed in Arlequin v3.11 [23]. The exact test of population differentiation was used with 1000 permutations and a significance level of 0.05.

## Results

### Analysis of *pfhrp2* and flanking genes

All 68 *P. falciparum* parasite isolates from Honduras were initially tested for the presence of *18S rRNA* and *msp2* and showed positive amplification, confirming the presence of good quality DNA. *Pfhrp2* and its flanking genes, MAL7P1.230 and MAL7P1.228, were found to be present in all parasite isolates.

### Analysis of *pfhrp3* and flanking genes

**Figure 2:**
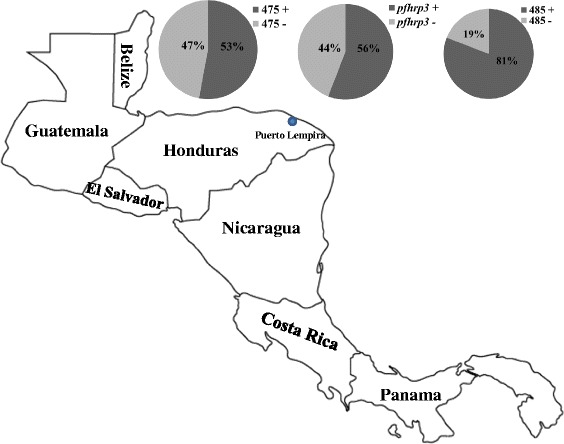
**Prevalence of deletions in** ***pfhrp3*** **and neighboring genes in** ***P. falciparum*** **isolates from Puerto Lempira, Nicaragua.** The map shows the location of Honduras in relation to neighboring countries in Central America. All parasite isolates analysed were found to be positive for *pfhrp2* and its flanking genes (not shown). The three pie charts shown illustrate the proportion of parasite isolates with deletions in *pfhrp3* and its neighboring genes. The percentages shown represent proportions of samples out of the total samples that were *18S rRNA*- and *msp2*-positive.

Thirty out of 68 isolates (44.1%) tested negative for the *pfhrp3* gene while 32 isolates (47.1%) showed deletion of the Mal13P1.475 gene that is located upstream of *pfhrp3* (Figure 2). Thirteen isolates had deleted the Mal13 P1.485 gene, located downstream of *pfhrp3* (Figure 2).

Thirty-four samples tested were positive for both *pfhrp3* and its flanking genes. One *pfhrp3*-positive isolate had deleted both flanking genes (Table 2). Forty-four percent of the parasite isolates tested were *pfhrp3*-negative and had additionally deleted either MAL13P1.475 or MAL13P1.485: 26.5% of the isolates were *pfhrp3*/MAL13P1.475 double-negative while 3% of the isolates were *pfhrp3*/MAL13P1.485 double-negative (Table 2). Approximately 15% of the parasite isolates had deleted all three genes (Table 2).

**Table 2:** **Deletion pattern of** ***Pfhrp3*** **and its flanking genes in two population clusters**

**MAL13P1.475**	***Pfhrp3*** **exon 1–2**	**MAL13P1.485**	**All samples, N (%)**	**Cluster 1**	**Cluster 2**
+	+	+	34 (50%)	19 (70.4%)	13 (35.1%)
+	+	-	0 (0%)	0 (0.0%)	0 (0.0%)
-	+	+	3 (4.4%)	1 (3.7%)	2 (5.4%)
+	-	-	2 (2.9%)	0 (0.0%)	2 (5.4%)
-	-	+	18 (26.5%)	6 (22.2%)	12 (32.4%)
-	-	-	10 (14.7%)	1 (3.7%)	8 (21.6%)
+	-	+	0 (0%)	0 (0.0%)	0 (0.0%)
-	+	-	1 (1.5%)	0 (0.0%)	1 (2.7%)
**Total**			**68**	27*	38*

The symbol ‘+’ indicates gene amplification while ‘-’ indicates no gene amplification after two attempts. N – number of samples. *A total of 65 samples were included in the population cluster analysis using Structure v2.3.3. Three samples were excluded because more than one allele was amplified in one or more microsatellite loci, indicating possible multiple strain infection. The columns labeled ‘Cluster 1’ and ‘Cluster 2’ show the absolute numbers (and percentage proportions) of samples with the indicated MAL13P1.475/ *Pfhrp3*/ MAL13P1.485 gene profiles that were assigned to each of the two parasite population clusters identified using Structure v2.3.3.

### Microsatellite genotyping and cluster analysis

**Figure 3:**
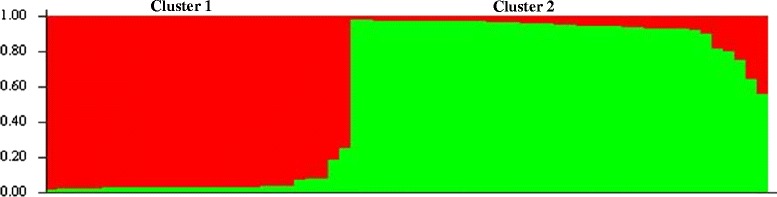
**Bayesian cluster analysis of** ***P. falciparum*** **singly-infected samples collected from Puerto Lempira, Honduras (N = 65).** The predicted number of likely clusters (*K*) for the samples was *K* = 2. Each color corresponds to a population cluster as classified by Structure v2.3.3, and each individual isolate is represented by a vertical bar. The Y axis represents the estimated proportion of membership of an individual to each population.

We performed neutral microsatellite genotyping and cluster analysis to determine if the population structure correlated with *pfhrp3*-deleted parasites. Structure analysis predicted the presence of at least two clusters of parasites (*K* = 2; Figure 3). Twenty-seven samples belonged to the first cluster (designated Cluster 1) while 37 were assigned to Cluster 2 (Figure 3). One isolate belonging to Cluster 1 was MAL13P1.475-negative (3.7%), six were MAL13P1.475/*pfhrp3*-negative (22.2%) and one had deleted all three genes (3.7%) (Table 2). Among the samples assigned to Cluster 2, two were MAL13P1.475-negative (5.3%), twelve isolates were MAL13P1.475/*pfhrp3*-negative (32.4%) while eight were MAL13P1.475/*pfhrp3*/MAL13P1.485 triple-negative (21.6%) (Table 2). In order to assess genetic differentiation due to population substructure, pair-wise F_st_ values were calculated, and found that Clusters 1 and 2 presented moderate genetic differentiation (0.12146; p-value < 0.05).

### Genetic diversity

*H*_*E*_, a measure of genetic variation at each locus, was between 0.2 and 0.7 with relatively few allelic forms of each marker (*A*) identified (Table 3). This is indicative of low genetic diversity in this parasite population. The number of alleles per locus in this population ranged from three to five (Table 4).

**Table 3:** **Microsatellite diversity for seven** ***P. falciparum*** **neutral microsatellite markers**

**Microsatellite marker name**	**TA1**	**Polyα**	**PfPk2**	**TA109**	**2490**	**C2M33**	**C3M69**
**Chromosome**	6	4	12	6	10	2	3
**Allelic size range, bp**	74–190	138–180	160–192	188–200	80–84	140–228	122–138
**No. of alleles per locus (** ***A*** **)**	4	5	3	2	2	5	5
PCR positivity*	60	65	64	62	60	64	65
***H***_***E***_	0.650	0.687	0.531	0.550	0.469	0.256	0.636

The microsatellite markers shown are described in the Methods section. bp – base pairs; PCR – Polymerase chain reaction. *PCR positivity indicates the number of samples that were successfully amplified for the indicated loci (out of a total of 68 samples). H_*E*_ – virtual heterozygosity where *H*_*E*_ = [n/(n–1)] × (1-Σp^2^i), and n is the number of samples. It is defined as the probability that a randomly chosen pair of alleles differ from each other.

**Table 4:** **Allelic frequencies at the seven** ***P. falciparum*** **microsatellite loci analysed**

**Microsatellite locus**	**Allele sizes (bp)**	**N**	**Frequency**
**TA1**	74	1	0.02
	140	34	0.52
	143	16	0.25
	190	9	0.14
	DNW	5	0.08
**Polyα**	138	2	0.03
	140	18	0.28
	142	1	0.02
	177	16	0.25
	180	28	0.43
**PfPk2**	160	13	0.2
	186	9	0.14
	192	42	0.65
	DNW	1	0.02
**TA109**	188	29	0.45
	200	33	0.51
	DNW	3	0.05
**2490**	80	15	0.23
	84	45	0.69
	DNW	5	0.08
**C2M33**	140	2	0.03
	220	1	0.02
	224	1	0.02
	226	56	0.86
	228	4	0.06
	DNW	1	0.02
**C3M69**	122	25	0.38
	124	2	0.03
	130	1	0.02
	136	7	0.11
	138	30	0.46

DNW – Samples that did not amplify an allele at the indicated locus.

## Discussion

Recent reports of *pfhrp2* gene deletions in natural *P. falciparum* isolates from Peru and other countries have highlighted the importance of molecular surveillance to detect these deletions because they could lead to false-negative diagnoses when using PfHRP2-based malaria RDTs [7,8]. Per common knowledge, this may be the first report to document the prevalence of *pfhrp2* and *pfhrp3* genes in natural *P. falciparum* isolates in Honduras. The approach taken in this study involved PCR amplification of the genes of interest (*pfhrp2* and *pfhrp3*) and their flanking genes in order to estimate the extent of deletion around *pfhrp2* and *pfhrp3*.

The data from this study showed no deletions of *pfhrp2* or its flanking genes (MAL7P1.228 and MAL7P1.230) in the samples tested, suggesting that the region on chromosome 8 spanning *pfhrp2* was intact in *P. falciparum* parasites in Puerto Lempira in 2008–2009. This data is similar to recent observations from French Guiana where no deletion of *pfhrp2* gene was found [24]. On the contrary, *pfhrp3* deletion was found in about 44% of the samples tested (Figure 2). Different combinations of deletion profiles for MAL13P1.475, *pfhrp3* and MAL13P1.485 were observed. The majority of *pfhrp3*-negative parasites were also either negative for the downstream gene MAL13P1.475 (~27%) or for both flanking genes (~15%) (Table 2). The highest prevalence of *pfhrp3*-negative parasites was reported in Peru, reaching as high as 70% [7]. In French Guiana, the prevalence of *pfhrp3*-negative parasite isolates was only 4.5% [24]. Previous research showed that *pfhrp3* deletions in the laboratory parasite line HB3, which is a Honduran parasite isolate, resulted from chromosomal deletions of a large genomic fragment in chromosome 13 [25,26]. Another study hypothesized that the deletion of *pfhrp3* in HB3 may have occurred as a result of the duplication and translocation of a region of chromosome 11 onto chromosome 13 [27]. It was not clear if *pfhrp3* was deleted before or after the HB3 strain was adapted to *in vitro* culture. Based on the results of this study, it can be speculated that *pfhrp3* deletion may have occurred in HB3 even before it was adapted to *in vitro* culture because these observations show it in naturally occurring parasite populations in Honduras.

Population structure analysis showed two major clusters of parasites. This low level of diversity is consistent with a recent study that showed similar results when comparing *P. falciparum* isolates from different municipalities in Honduras (including Puerto Lempira) and Nicaragua [28]. In this study, *pfhrp3* deleted parasites were observed in both clusters. In the study conducted by Larranaga *et al.*, Bayesian cluster analysis predicted that most of the isolates collected from mainland Honduras belonged to a single cluster [28]. However, it is possible that the outcome of the analysis may have been different if cluster analysis had been performed on the samples from Honduras alone rather than including isolates from Nicaragua [28]. Furthermore, these results indicate that there is still some level of substructure present (Figure 3) in this population. Nevertheless, a greater number of samples including complete haplotype information would be needed to draw further conclusions. The area in which Puerto Lempira is located accounted for nearly 68% (419/610) of *P. falciparum* cases reported in Honduras in 2008 (the year in which sample collection started for this study).

One limitation of this study is that all samples were collected from one location and only within a one-year period; further testing would need to be conducted on *P. falciparum* isolates collected from various geographical regions of Honduras in order to confirm that the current findings are applicable to the entire country. It should be noted, however, that most of Honduras reports very few malaria cases annually and Puerto Lempira is one of the areas where RDTs need to be used for programmatic purposes due to limited access to microscopy [3]. As such, PfHRP2 surveillance in Central America should continue in order to monitor the pattern of distribution of *pfhrp2* and *pfhrp3* deletions.

Central America has relatively low levels of malaria transmission compared to most of the malarious regions of South America. RDTs are especially useful in areas where there is limited access to microscopy for malaria surveillance and elimination programs. In Honduras, both *P. falciparum* and *P. vivax* transmission occur and although microscopy is used as a primary diagnostic test for case management, RDTs are useful in remote areas where access to microscopic diagnosis is limited. To detect both *P. falciparum* and *P. vivax*, RDT combination tests that can detect both species are recommended. In this context, these finding suggests that *pfhrp2*-negative parasites have not evolved or spread in Honduras and that PfHRP2-based RDTs can still be considered for use in this region as part of the combination tests for *P. falciparum* and *P. vivax* diagnosis.
